# The Cingulate Island Sign is Useful for a Differential Diagnosis of Early-Onset Alzheimer's Disease and Dementia with Lewy Bodies: A 99mTc-ECD SPECT Study

**DOI:** 10.14789/jmj.JMJ22-0018-OA

**Published:** 2022-09-09

**Authors:** MOTO NISHIGUCHI, TOSHIKI TAKAYAMA, KOJI KASANUKI, TSUNEYOSHI OTA, NOBUTO SHIBATA, YOSUKE ICHIMIYA, HEII ARAI

**Affiliations:** 1 Department of Psychiatry and Behavioral Science, Juntendo University Graduate School of Medicine, Tokyo, Japan; 1 Department of Psychiatry and Behavioral Science, Juntendo University Graduate School of Medicine, Tokyo, Japan; 2 Department of Neuropsychiatry, St. Marianna University School of Medicine, Kanagawa, Japan; 2 Department of Neuropsychiatry, St. Marianna University School of Medicine, Kanagawa, Japan; 3 Alzclinic Tokyo, Tokyo, Japan; 3 Alzclinic Tokyo, Tokyo, Japan

**Keywords:** early-onset dementia, early diagnosis, differential diagnosis, SPECT

## Abstract

**Introduction:**

Early-onset dementia is fast-progressing compared with late-onset dementia, with major clinical characteristics including prominent focal cerebral symptoms. Given its economic and psychological implications, proper diagnosis and treatment at an early stage is essential. In the present study, the authors conducted a retrospective study to evaluate the usefulness of various numerical indices (including CIScore calculated by eZIS, cerebral blood flow SPECT analysis software) in the differential diagnosis of early-onset dementia.

**Materials and Methods:**

This study involved patients with early-onset and mild dementia who were receiving ambulatory care at our outpatient department specializing in Alzheimer's disease (14 MCI patients, 16 AD patients, and 16 probable/possible DLB patients). ROC analysis was performed for each SVA numerical index calculated by eZIS to calculate AUC. For the AD and DLB groups, correlation between the CIScore and MMSE was assessed.

**Results:**

When SVA-A (severity) was used to differentiate AD from MCI and DLB from MCI, the respective AUC values were 0.960 and 0.911. When CIScore was used to differentiate AD from DLB (threshold value: 0.225), the obtained AUC value was 0.941, and the accuracy, sensitivity, and specificity were 90.6%, 87.5%, and 93.7%, respectively. No significant correlation was observed between the MMSE and CIScore scores in these disease groups.

**Conclusion:**

The results of this study have suggested that the SVA-A is a useful index for evaluating the conversion from MCI to either early-onset AD or DLB, and that the CIScore is useful for differentiating AD from DLB in both late-onset and early-onset dementia cases.

## Introduction

Early-onset dementia is a term used to describe any form of dementia that develops in people aged 64 or below. Epidemiological research estimates that approximately 40 thousand people have this condition in Japan. The most common cause is vascular dementia (VD), which is followed by Alzheimer's disease (AD), head trauma, and dementia with Lewy bodies (DLB)^1)^. Compared with late-onset dementia, early-onset dementia is fast-progressing and clinically characterized by prominent focal cerebral symptoms. Given its economic and psychological implications, proper diagnosis and treatment at an early stage is essential.

Neuropsychological tests, such as Mini-Mental State Examination (MMSE)^2)^, are the primary means of diagnosing dementia. However, recent advances in cerebral function measurement technology have made it practicable to employ brain imaging analysis as an auxiliary measure for more accurate diagnosis. Technologies that are typically utilized for brain imaging include magnetic resonance imaging (MRI) and single photon emission computed tomography (SPECT). Clinically, detecting minute brain atrophy, a characteristic symptom of early-stage AD, requires high skill and long time. With the aim of easily and quantifiably identifying atrophied lesions in the hippocampus and parahippocampal gyrus in MR imagery, the Voxel-Based Specific Analysis System for Alzheimer's Disease (VSRAD)^3)^ has been recently developed and is now being used in medical practice. However, patients with early-onset dementia often have hippocampus/parahippocampal gyrus atrophies that are fairly inconspicuous, and it is not uncommon for VSRAD-based indices to fall within the normal range. At the stage of mild cognitive impairment (MCI), which is considered to be the prodromal phase of AD, known cerebral blood flow findings include a decrease in blood flow in the parietal lobe, precuneus, and posterior cingulate gyrus. Since even AD patients tend to display high blood circulation in the precuneus and posterior cingulate gyrus, it is difficult to macroscopically detect a decrease from early stages. An easy Z-score imaging system (eZIS) was developed to compare the normal blood flow of healthy people with that of AD patients^4-6)^ and is now being used for aiding AD diagnosis.

Depressiveness, hallucinations, and delusions are frequently observed symptoms of DLB^7)^. However, presenile DLB patients are often falsely diagnosed with depression and/or schizophrenia due to these diseases sharing similar symptoms^8)^. Impaired cognitive functions and psychiatric symptoms derived from DLB can be effectively treated with donepezil^9)^. Therefore, early diagnosis is more crucial for DLB than for AD because it can significantly affect the prognosis. Moreover, predispositions of DLB patients, such as high sensitivity of antipsychotics, mean that differential diagnosis is critical. In DLB, sugar metabolism and blood flow decrease in the occipital lobe. Additionally, 18F- FDG PET imaging reveals a cingulate island sign (CIS), a finding that sugar metabolism is relatively retained in the posterior cingulate gyrus in DLB as opposed to AD^10)^. According to the Clinical Diagnostic Criteria for DLB (2017 Revised Edition), the CIS is regarded as one of the major biomarkers^11)^. Furthermore, a recent study has reported that DLB patients exhibit CIS findings are in cerebral blood flow SPECT using 99mTc-ECD, as compared with AD patients^12)^. Another study has demonstrated the high effectiveness of CIScore (a reference index using CIS in eZIS) in differentiating AD from DLB^13)^.

In the present study, we retrospectively evaluated the usefulness of various numerical indices calculated by eZIS (cerebral blood flow SPECT analysis software) in differentiating types of early-onset dementia.

## Materials and Methods

The present study was approved by the Ethics Committee of Juntendo University Hospital (No. JHS 17-0017) and was conducted with the informed consent of the subjects and their families in writing.

The study involved patients with early-onset and mild dementia who were receiving ambulatory care at our outpatient department specializing in Alzheimer's disease. The patient characteristics are summarized in Table 1. The subjects comprised 14 MCI patients (11 men and 3 women), 16 AD patients (11 men and 5 women), and 16 probable/possible DLB patients (6 men and 10 women). The MCI patients were diagnosed in accordance with Petersen's Criteria 2004, were 51 to 69 years old (mean: 58.4; standard deviation 6.3), and their MMSE score ranged from 22 to 30 (mean: 27.4; standard deviation: 2.3). The AD patients were diagnosed in accordance with DSM-IV-TR, were 47 to 68 years old (mean: 58.3; standard deviation: 5.7), and their MMSE score ranged from 4 to 26 (mean: 20.4; standard deviation: 6.1). The DLB patients were diagnosed in accordance with the 3rd International WS DLB, were 54 to 69 years old (mean: 61.6; standard deviation: 3.8), and their MMSE score ranged from 5 to 26 (mean: 16.7; standard deviation 6.6).

**Table 1:** Demographic data

Characteristic	MCI (n=14)	AD (n=16)	DLB (n=16)
Male / Female	11 / 3	11 / 5	6 / 10
Age (mean±SD)	58.4±6.3	58.3±5.7	61.6±3.8
MMSE score (mean±SD)	27.4±2.3	20.4±6.1	16.7±6.6

All subjects underwent head SPECT at our Radiology Department. For SPECT, TOSHIBA GCA 9300 A, and SIEMENS Symbia_E, and S were used. Additionally, a fan beam collimator was used. A tracer (99mTc-ECD) was administered to all participants at a supine position, with the eyes closed. The parameters of SPECT imaging were as follows: matrix size: 128×128; step angle: 4 degrees; view: 90; rotation time: 1min; and rotation: 16.

**Figure 1:**
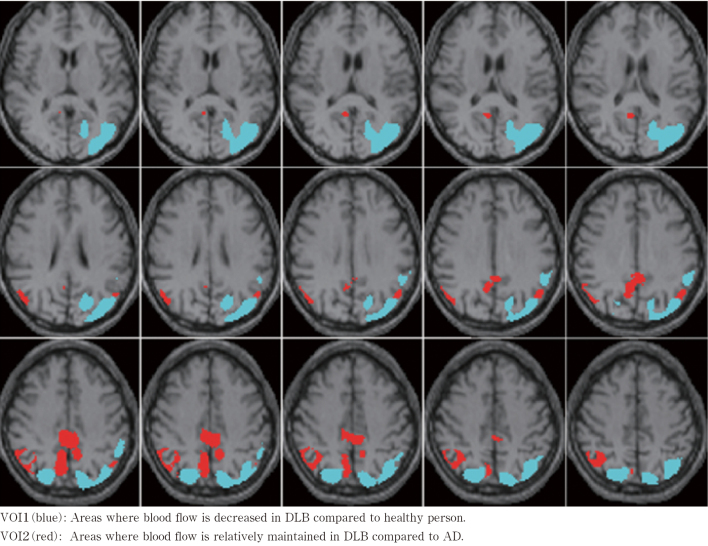
Examples of VOI1 and VOI2

Automatic analysis using eZIS (version 1.1.0) was performed to set a volume of interest (VOI). With eZIS, specific VOI analyses (SVA-A and SVA-B) were performed. The VOI of SVA-A is the area with significantly decreased blood flow (p < 0.001) in the 99mTc-ECD image of an incipient Alzheimer's dementia patient with mildly impaired cognitive functions (amnestic MCI), which is comparatively analyzed against the 99mTc-ECD image of a healthy person. The image includes the posterior and anterior cingulate gyrus and a part of the vertex. As numerical indices, severity (degree of blood flow decrease in the VOI), extent (percentage of area with decreased blood flow in the VOI), and ratio (ratio of area with decreased blood flow in the VOI and that in the entire brain) are calculated. The VOI of SVA-B is the area with significantly decreased blood flow (p < 0.05) in the 99mTc-ECD image of a DLB patient compared with that of a healthy person. When this area VOI1 (mainly the posterior head) is subtracted from the VOI of the aforementioned SVA-A VOI, the VOI2 (mainly the posterior cingulate gyrus) is set (visualization in Figure 1). The sum of the Z scores in the area with decreased blood flow obtained from these two VOIs are used to calculate the CIScore (CIScore = sum of the Z scores in the decreased blood flow area obtained from the VOI2/sum of the Z scores in the decreased blood flow area obtained from the VOI1). In a multi-center study so far, the normal Z score value has been reported as severity ≤ 1.19, extent 14.2 ≤ 2%, and ratio ≤ 2.22^14)^. The MCI, AD, and DLB groups were analyzed by eZIS SVA calculate various numerical indices (severity, extent, ratio, and CIScore). Significant differences in the Z scores of the VOI1 and VOI2 were verified between the groups. Receiver operating characteristics (ROC) analysis was performed to calculate the area under the ROC curve (AUC). Additionally, the correlation between the CIScore and MMSE was assessed for the AD and DLB groups.

## Results

Table 2 lists the numerical indices of all the groups, their means, and standard deviations. Mann-Whitney U test was performed for the numerical indices of each group, with p < 0.05 being the level of significance.

**Table 2:** SVA index (mean±SD) in each group

	Severity	Extent	Ratio	CIScore	VOI-1	VOI-2
MCI	1.53^†, ‡^ ±0.33	26.27 ±13.80	4.29 ±2.40	0.227 ±0.130	8677 ±2478	1771 ±580
AD	3.38^†^ ±1.31	68.72 ±20.41	5.84 ±2.73	0.358 ^§^ ±0.159	15159 ±7607	4826 ±2173
DLB	3.19^‡^ ±1.27	62.82 ±23.97	3.88 ±1.25	0.173 ^§^ ±0.057	24616 ±9125	4226 ±1971

†, ‡, §： significant difference

**Figure 2:**
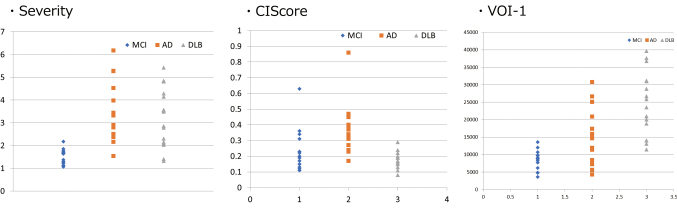
Boxplot in each group

**Figure 3:**
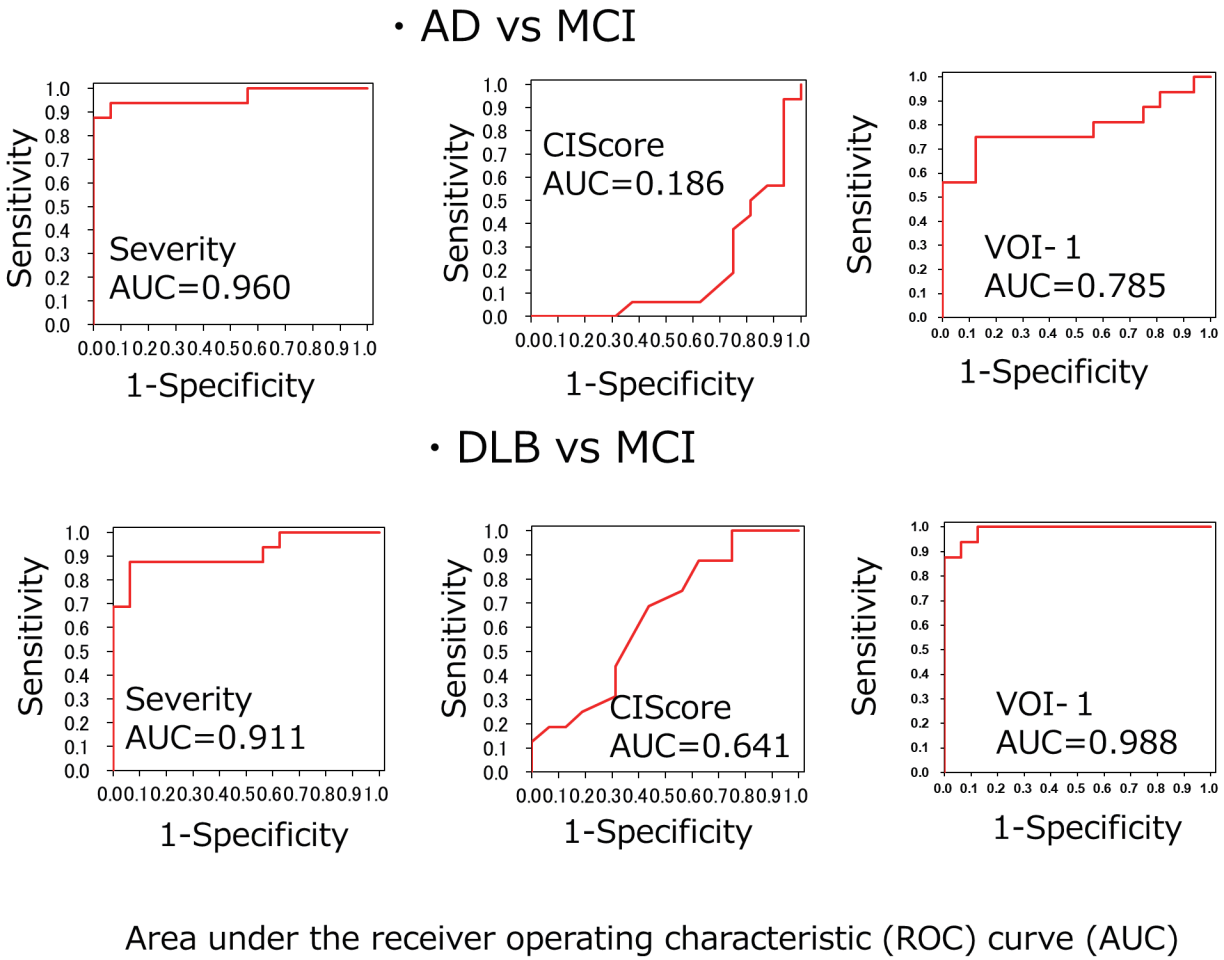
ROC analysis (AD vs MCI, DLB vs MCI)

**Figure 4:**
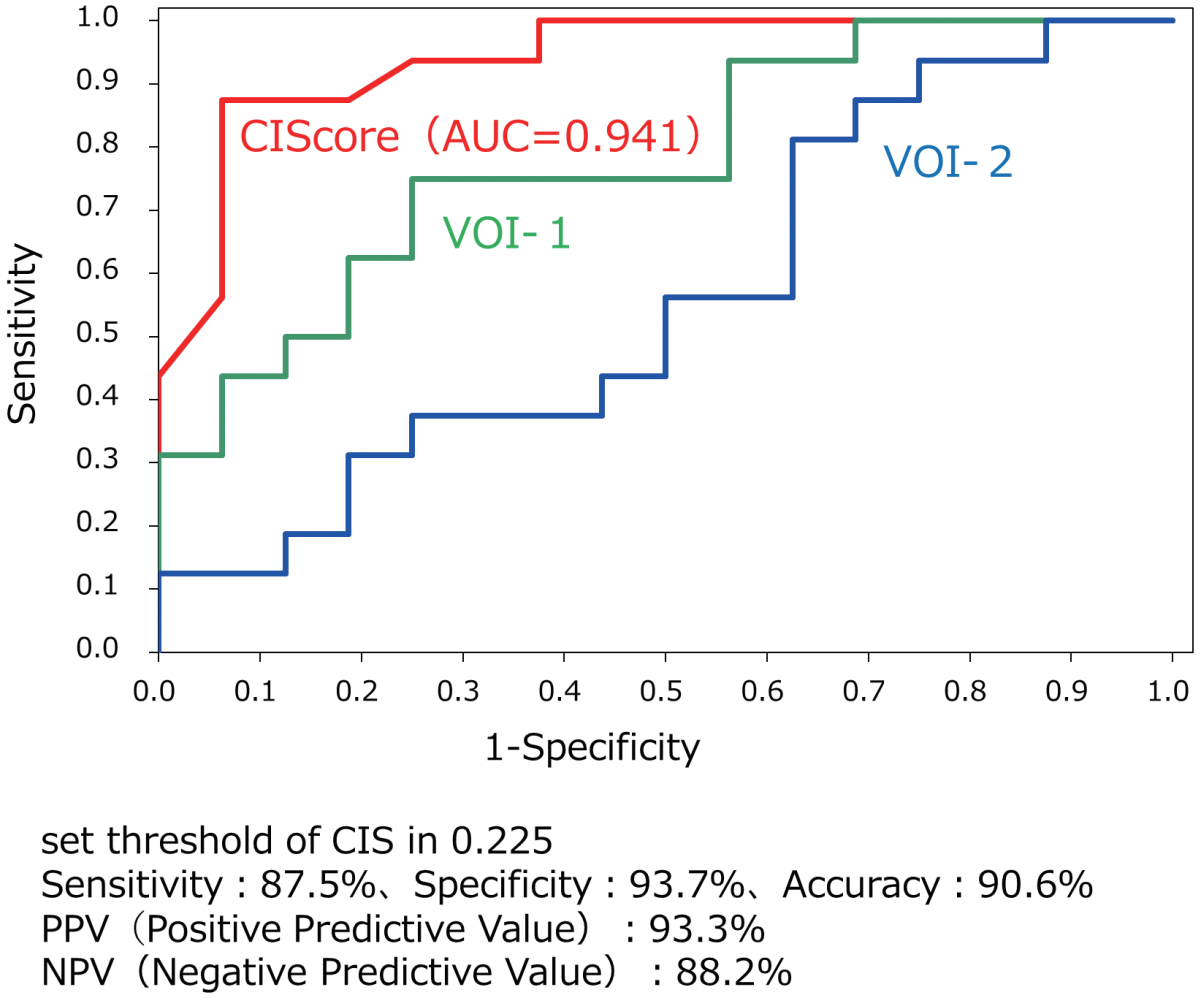
ROC analysis (AD vs DLB)

**Figure 5:**
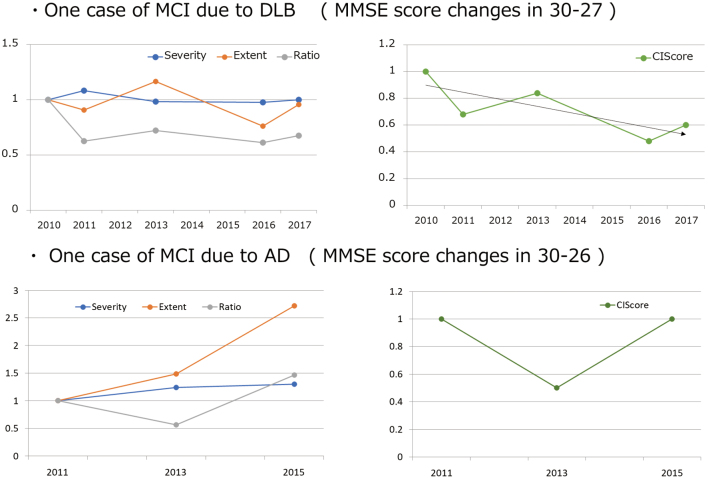
Rate of change from the testing first year (write an initial value for 1)

**Figure 6:**
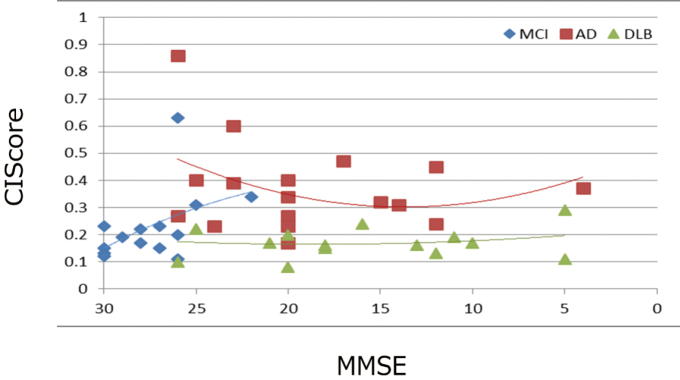
The correlation between MMSE and CIScore in each group

In the MCI group, all severity, extent, and ratio values exceeded the normal range. When the MCI group was compared with the AD and DLB groups, both the severity and extent values were significantly high, and the ratio value was not significantly different. Box plots of the Severity and the CIScore are shown in Figure 2. When severity was used for differentiating MCI from AD and DLB, the respective AUC values were 0.960 and 0.911. Likewise, when the CIScore and VOI1 were used, the respective AUC values were 0.186, 0.641, 0.785, and 0.988 (Figure 3). When the CIScore was used for differentiating AD from DLB, the obtained AUC was 0.941. When the threshold value was set at 0.225, the accuracy, sensitivity, and specificity were 90.6%, 87.5%, and 93.7% (Figure 4). When chronological changes of the SVA-A and CIScore in the MCI due to AD and MCI due to DLB cases were tracked, the chronological changes of the CIScore tended to decline (Figure 5). In all disease groups, no significant correlation between the CIScore and MMSE was observed (Figure 6).

## Discussion

Compared with the MCI group, both the AD and DLB groups exhibited significantly increased severity and extent, and no significant difference was observed in the ratio. The obtained AUC also had high severity and extent, thus suggesting that severity and extent were useful indices for evaluating the conversion of MCI into other diseases in both the AD and DLB groups. The obtained AUC indicates that severity is more useful for differential diagnosis. The severity and extent significantly increased in the AD and DLB groups. In other words, the significant increase in severity reflected the conversion and aggravation into AD and DLB. In contrast, the ratio was not significantly different between the groups, meaning that the ratio was almost constant regardless of the severity. These trends are consistent with previous studies using eZIS that involved healthy elderly people and mild to serious AD patients (Matsuda et al.)^14-16)^.

The above results suggest that severity and extent are useful indices for evaluating the conversion of MCI into AD and DLB. However, no significant difference was observed between the AD and DLB groups. Therefore, use of the VOI1, the volume of interest of DLB, is the most effective means of differentiating MCI from DLB.

In contrast, it was suggested that CIScore is not suitable for evaluating conversion from MCI to AD and DLB. The VOI1 is the volume of interest of DLB, and the VOI2 is the area that remains after the VOI1 is subtracted from the SVA-A, the volume of interest of AD. Independently, these indices reflect the quantity of blood flow decrease in the respective areas. Significant difference was observed, suggesting the progression from MCI to AD or DLB. However, the CIScore, the ratio of these indices, did not show significant difference. The MCI group includes “due to AD” and “due to DLB”, thus implying the possibility that characteristics of both diseases start developing from the MCI stage. Further research needs to be conducted to compare patients with healthy people and longitudinally evaluate indices. In the case of poor blood flow reduction, it is assumed that the above results were obtained because the relative difference increased by taking the ratio.

The CIScore was highly effective in differentiating early-onset AD and DLB. In a previous study, Imabayashi et al. used the CIScore for differentiating late-onset AD and DLB^13)^ (AUC0.882). The present study achieved a higher differentiating capability. This may reflect the fact that compared with late-onset dementia, blood flow decrease is more prominent in the VOI than in the area of brain atrophies in morphological imagery in early-onset dementia., and higher values tend to appear in the SVA^16)^. Therefore, this suggests that it may be useful for differentiation in early-onset dementia. However, the fact that the clinical diagnosis also referred to the cerebral blood flow SPECT imaging findings may be the cause of the particularly high sensitivity and specificity levels.

In the MCI due to DLB group, the CIScore tended to decline over time in a single case. In other words, the CIS findings increased. Iizuka et al. have reported that the CIS findings increased as the MMSE score decreased down to around 22. However, below the score of 22, the CIS findings disappeared^17)^. In the present study, where the final MMSE score was 27, the results were consistent with the tendency reported by them. Therefore, in the early-onset DLB group, the MMSE was predicted to correlate with the CIScore reflecting changes in blood flow. However, no apparent correlation was observed as a whole. Iizuka et al. have argued that the disappearance of the CIS findings is related to the progression of AD pathology, thus explaining the changes in the CIS findings by the chronological changes of the posterior cingulate cortex and precuneus plus cuneus^17)^. In general, the neuropathological findings of DLB include numerous Lewy bodies in the cerebral cortex and AD pathological findings such as senile plaques and degraded neurofibrils (common form). In contrast, early-onset cases often exhibit only Lewy lesions without showing any AD lesions (pure form)^18)^. Moreover, just like atrophies and tissue denaturation in the cerebral cortex differ between AD and SDAT, the characteristics of DLB were maintained in early-onset cases from MCI to more advanced conditions. Consequently, no correlation between the MMSE and CIScore was observed.

For differentiating early-onset dementia cases, eZIS was used. When severity was used for differentiating MCI from AD and DLB, the AUC values were 0.960 and 0.911, respectively. The CIScore was used for differentiating AD from DLB, and the threshold value was set at 0.225. The resultant AUC was 0.941, and the accuracy, sensitivity, and specificity were 90.6%, 87.5%, and 93.7%, respectively. The SVA-A was suggested to be useful for evaluating the conversion of MCI into both early-onset AD and DLB. Furthermore, the CIScore was suggested to be highly effective in differentiating AD from DLB not only in late-onset dementia but also early-onset dementia cases. It is important to understand the characteristics of each index and apply it to clinical practice.

## Funding

No funding was received.

## Author contributions

TO and HA were the doctors in charge of these cases and collected the data. MN analyzed and interpreted the data and wrote the manuscript. TT, KK, NS and YI provided guidance on manuscript writing. All authors have read and approved the final manuscript.

## Conflicts of interest statement

The authors declare that there are no conflicts of interest.
